# Low-Frequency Ultrasound Assisted in Improvement in Cell Development and Production of Parasporal Crystals from *Bacillus thuringiensis* HD1

**DOI:** 10.3390/insects16050507

**Published:** 2025-05-09

**Authors:** Sufen Cui, Kaihui Shen, Shiqi Xiong, Xiao Li, Yue Wang, Xueqing Geng, Yujie Lu

**Affiliations:** 1 School of Grain Science and Technology, Jiangsu University of Science and Technology, Zhenjiang 212004, China; 231211804103@stu.just.edu.cn (K.S.); 18651409127@163.com (S.X.); 17851003951@163.com (X.L.); 19953996098@163.com (Y.W.); luyjlyj71@just.edu.cn (Y.L.); 2 Department of Resources and Environment, School of Agriculture and Biology, Shanghai Jiao Tong University, Shanghai 200240, China; xqgeng@sjtu.edu.cn

**Keywords:** low-frequency ultrasound, *Bacillus thuringiensis*, parasporal crystals, insecticidal activity, *Sitophilus zeamais*

## Abstract

*Bacillus thuringiensis* is widely utilized as a microbial insecticide due to its production of parasporal crystals during the spore-forming stage. However, lower fermentation efficiency coupled with elevated production costs limit its broad application. Low-frequency ultrasound (LFU) has been employed in the fermentation industry to enhance microbial growth and metabolism. In this study, the effect of LFU on the growth of *B. thuringiensis* HD1 and the yields of parasporal crystals was investigated. The maximum biomass accumulation of *Bacillus thuringiensis* and parasporal crystal production yield were achieved following low-frequency ultrasonic (LFU) treatment applied during the logarithmic growth phase (18 h of cultivation) under optimized parameters: a frequency of 40 kHz, a power output of 176 W, and an irradiation duration of 45 min. Under optimal conditions, LFU significantly increased the cell membrane permeability and secretory inositol, favoring cell growth and parasporal crystal production. FESEM/CLSM and TEM analyses visually displayed the changes in cell morphology. In addition, the germination rate of spores was increased after LFU treatment, which further confirmed the positive effect of LFU on the growth of *B. thuringiensis*. Compared to the control, parasporal crystals harvested under LFU exhibited significant modifications in their physicochemical characteristics; the particle size increased, the surface electronegativity intensified, and there was a morphological transition from spherical to cubic geometry. Importantly, the parasporal crystals exhibited strong insecticidal activity against *S. zeamais* adults, a typical stored-product insect pest, with an LC_50_ of 10.795 mg/g on day 14 and a Kt_50_ of 4.855 days at a concentration of 30 mg/g. These findings will provide new insights into the product development and application of *B. thuringiensis* in the future.

## Simple Summary

*Sitophilus zeamais* Motschulsky (Coleoptera: Curculionoidea), a major pest of cereal grains, leads to great losses both in the quality and quantity of crops throughout the world. Although traditional methods such as fumigation could effectively control this pest, various problems, including environmental pollution, food contamination and insecticidal efficiency, are becoming more and more serious. Developing new biological protective agents based on microorganisms could be a good alterative choice. *Bacillus thuringiensis*, a Gram-positive bacterium, is considered to be a potential microbial insecticide due to its production of parasporal crystals. So, the production of parasporal crystals is critically important for the application of this kind of insecticide. In this paper, the low-frequency ultrasonic (LFU) treatment was used to improve the efficiency of the development of *B. thuringiensis* and the production of parasporal crystals; then, their physicochemical properties, such as cell morphology, parasporal crystals particle shape, and spore germination, were investigated by FEFEM, CLSM, and other testing methods. Correspondingly, the pesticide activity of parasporal crystals obtained against *S. zeamais* was examined. The results showed that LFU could significantly improve the development of *B. thuringiensis* cells under appropriate parameters, and the productivity of parasporal crystals was synchronously increased. The product obtained also showed excellent insecticide activity against *S. zeamais*. This study might provide good insights for the further development and application of this valuable organism.

## 1. Introduction

*Bacillus thuringiensis* (Bt), a member of the *Bacillus cereus sensu* lato (Bcsl) complex, is currently recognized as the most widely utilized microbial insecticide due to its ability to produce parasporal crystals, such as pore-forming Cry toxins with or without the cytolytic Cyt toxin [1]. Bt demonstrated remarkable antibacterial and pesticidal properties, along with being conducive to environmental sustainability. It has been reported that Cry proteins are effective against various kinds of insects, such as Lepidoptera, Coleoptera, Diptera, and Hymenoptera [2]. Once insects consume food containing Bt crystals, the alkaline environment of their gut dissolves these crystals, releasing Cry proteins. These proteins can bind to specific receptors on the intestinal tract of the insect, resulting in perforation of the intestinal cell membranes and subsequent lysis of the intestinal cells [3]. This process ultimately leads to a cessation of feeding and death of the insect. Furthermore, Bt and its associated spore crystals exhibit very low toxicity to non-target organisms, including humans and other vertebrates. In addition to its direct application as a pesticide, the gene encoding of Cry proteins has been extensively utilized in the development of transgenic crops to enhance their resistance to insect pests [4].

Low-frequency ultrasound (LFU), a mechanical wave with a frequency range from 20 to 100 kHz, has garnered interest due to its potential for use in food fermentation processes for over 20 years. It could enhance the heat and mass transfer of substances, thereby promoting microbial growth and metabolite synthesis [5,6,7]. Early studies have found that LFU treatment at 24 kHz enhances the mycelium growth of *Eremothecium ashbyii* and consequently increases riboflavin production [8]. Ultrasonic treatment at 28 kHz enhanced the efficiency of *Lactobacillus paracasei* in the production of yogurt peptides for immediately activating extracellular enzyme activity [9].

In this paper, LFU was employed to promote *B. thuringiensis* fermentation for parasporal crystals. The effect of ultrasonic frequency, power, time, and the application of ultrasound at the microbial growth stage on the physicochemical characteristics of *B. thuringiensis* and parasporal crystal production was investigated. Inositol is an essential metabolic molecule for spore formation, which is crucial to the processes of signal transduction, structural composition, and the stress response [10,11]. So, the content of inositol of *B. thuringiensis* cells was also determined. In addition, the effect of parasporal crystals obtained against *Sitophilus zeamais* Motschulsky (Coleoptera: Curculionoidea), a major pest of cereal grains, was also studied [12,13]. This work might provide novel strategies for enhancing the germination efficiency of *B. thuringiensis*, thereby facilitating more effective industrial applications. Additionally, the insights gained from this research could be conducive to developing a deeper understanding of the underlying mechanisms of microorganism fermentation assisted by ultrasound.

## 2. Materials and Methods

### 2.1. Materials

Luria–Bertani (LB) agar was purchased from Bioway Technology Co., Ltd., Shanghai, China; *Bacillus thuringiensis* subsp. *kurstaki* (HD1) was purchased from Mingzhoubio technology Co., Ltd., Ningbo, China. Lysozyme was purchased from Hongrun Baoshun Technology Co., Ltd., Beijing, China.

### 2.2. Effect of Ultrasound on B. thuringiensis HD1 Cells

#### 2.2.1. *B. thuringiensis* Stains and Culture

*B. thuringiensis* HD1 was activated by inoculating 50 µL of bacterial suspension into 100 mL of LB liquid medium, and incubated at 30 °C and 180 r/min for 24 h, prior to growth curve plotting and subjecting the strain to ultrasonic treatment. A total of 10 mL of 10^6^ cfu/mL bacterial suspension was inoculated into 50 mL of sterile LB medium and incubated at 30 °C and 180 r/min. The absorbance of culture medium was determined at 600 nm at intervals of 2 h for 32 h using a spectrophotometer (T6-1650F, Beijing puxi, instruments Co., LTD, Beijing, China). The growth curve of *B. thuringiensis* HD1 was plotted, and its growth phases were identified accordingly.

#### 2.2.2. Ultrasound Treatments

Ultrasonic equipment (KQ-400DE, Kunshan ultrasonic instruments Co., LTD, Kunshan, China) with a fixed-frequency (40 kHz) and a continuous ultrasound mode was employed to enhance the growth of *B. thuringiensis* HD1. The effects of the ultrasonic power (88, 176, 352, 440 W), ultrasonic treatment duration (15, 30, 45, 60, and 75 min), and bacterial growth stage (cultured for 8, 18, and 26 h) in a water bath (15 L) at 30 °C on the growth of the strain was evaluated after 32 h of incubation. Then, the cells and supernatant were obtained by centrifugation at 6000 rpm and 4 °C for 15 min for subsequent experiments. The untreated strain was used as the control.

### 2.3. Preparation of Parasporal Crystals

The extraction of parasporal crystals was performed according to a previous method (slightly modified) [14]. A total of 1 mL of 500 μg/mL of lysozyme solution was added to the 50 mL bacterial culture mentioned above and mixed thoroughly. After 20 min, the lysed cells were acquired, immediately frozen at −20 °C for 30 min, and subsequently heated in a water bath at 40 °C for 15 min. Through three repeated cycles of freezing and thawing, the crude spore–crystal protein pellet was obtained after centrifugation (4 °C and 12,000 r/min for 20 min) and three rounds of washing while suspended in 1 M NaCl containing 0.01% triton X-100. The pellet was then suspended in a 50 mL centrifuge tube containing a saline solution. A ratio of 10% petroleum ether was added to the tube and mixed thoroughly. The suspension was sonicated at 100 W for 30 min to disperse clumps, then left to stand in a fume hood for 12 h and centrifuged at 6000 rpm for 10 min, and the crystal protein precipitate was obtained. After being washed twice with distilled water, the crystal protein was dried in a vacuum oven at 40 °C. The yield of crystal protein was recorded.

### 2.4. Determination of Cell Membrane Permeability

After 18 h of culturing, *B. thuringiensis* was treated with LFU (176 W; 40 kHz; 37 °C; 0, 15, 30, 45, 60, and 75 min), and then transferred to an incubator for continued incubation. Six hours later, the OD_260_ and OD_280_ values were determined [15]. Each treatment was repeated three times.

### 2.5. Characterization of B. thuringiensis Cell Morphology

The properties of *B. thuringiensis* cell morphology under the optimal ultrasound parameters were analyzed using the following technologies.

Field Emission Scanning electron microscopy (FESEM) (Carl Zeiss, Merlin Compact, Oberkochen, German) was used to investigate the morphology of *B. thuringiensis* cells and the crystal proteins. The example was prepared for FESEM analysis according to previous reports [1,16]. Briefly, the *B. thuringiensis* cells were washed with PBS (pH = 7.5), pre-fixed with 2.5% glutaraldehyde for 12 h, and washed with PBS again. Then, serial dehydration was carried out using 50%, 70%, 80%, 90%, and 100% ethanol. The fixed cells were dried and coated with gold, and the morphology of the samples was observed.

A laser confocal scanning electron microscope (CLSM) (ZEISS LSM900, Oberkochen, Germany) was used to observe *B. thuringiensis* cells following the methods used in previous studies with slight modifications [16,17,18,19]. The cells were fixed with 2.5% glutaraldehyde, washed and re-suspended in 0.01 M PBS, incubated with 5 μg/mL propidium iodide for 15 min, then washed with 0.01 M PBS. Finally, 5 μL of cell suspension was dropped onto the glass slide, covered with coverslips without creating bubbles, and observed using CLSM.

### 2.6. Characterization of B. thuringiensis Parasporal Crystals

The physicochemical properties of parasporal crystals obtained under the optimal ultrasound parameters were observed using FESEM, Fourier Transform Infrared (FTIR) spectra, and a zeta potential analyzer.

FESEM was used to examine the morphological properties of parasporal crystals, after sputter-coating the samples with a Au-Pd alloy (80:20 wt%) to enhance surface conductivity.

The Fourier Transform Infrared (FTIR) spectra of parasporal crystals were in the range 4000–500 cm^−1^, obtained using an FTIR spectrophotometer (Bruker, Invenio, German) via the potassium bromide (KBr) pellet method [6].

The surface charges of the parasporal crystals were determined using a zeta potential analyzer (Malvernzeta Asizernano ZS90, Malvern Instruments LTD, Worcestershire, UK).

### 2.7. Determination of Germination Activity of B. thuringiensis Spores

According to the methods of TALUKDER (with slight modifications), the spore germination was examined [20]. *B. thuringiensis* was incubated until the sporulation frequency reached 90–95%, and then harvested and stored at 4 °C. The spore germination was examined using an optical microscope. A total of 30 mL of spore suspension was diluted in sterilized water to OD_600_ ≈ 1.0, incubated at 75 °C for 15 min, and cooled; then, it was treated with different durations of LFU (176 W; 40 kHz; 37 °C; 0, 15, 30, 45, 60, and 75 min) and incubated in the incubator for 60 min at 37 °C and 180 r/min. The spore solution was observed, and the OD_600_ value was measured at 0, 15, 30, 45, and 60 min using an ultraviolet spectrophotometer (T6-1650F, PERSEE, Beijing, China) [21]. Each treatment was replicated three times. After ultrasonic treatment, peacock and sand yellow staining, and microscopy, were performed to observe the spore germination.

The germination rate was calculated by Equation (1):

(1) $${Reduction\;rate\;of\;OD}_{600}(\%)=\frac{{OD}_{0}-{OD}_{t}}{{OD}_{0}}\times 100\%$$

where *OD_t_* is the absorbance value at 600 nm of the *Bacillus thuringiensis* culture at time *t*, and *OD*_0_ is the absorbance value at 600 nm of the initial spore suspension.

### 2.8. Analysis of Inositol Concentration

The content of inositol in *B. thuringiensis* fermentation supernatant was analyzed using a UHPLC system. UHPLC analyses were conducted using Ultra-Performance Liquid Chromatography–Quadrupole Time-of-Flight Mass Spectrometry (Ultra-HPLC-TOF-MS) (G2-XS, Waters, Milford, CT, USA) with an Acquity HSS T3 column (2.1 × 100 mm, 1.8 μm) coupled to an Acquity UPC^2^/Xevo G2-XS QTOF mass spectrometer (Waters, Milford, CT, USA). Mobile phase A consisted of 0.1% formic acid in water, and mobile phase B consisted of 0.1% formic acid in acetonitrile. The elution procedure parameters were as follows: 99% A, 1% B; 85% A, 15% B; 70% A, 30% B; 50% A, 50% B; 100% B; 100% B; 99% A, 1% B; and 99% A, 1% B for 2, 3, 9, 11, 15, 18, and 20 min, respectively. A total of 3 μL of sample was injected into the LC-QTOF-MS system and electro-sprayed in negative mode. The full scanning range was from 50 to 1500 m/z, and the interval time was 0.2 s.

### 2.9. Insecticidal Activity of B. thuringiensis Parasporal Crystals

#### 2.9.1. Insects

*S. zeamais* were reared in an environmentally controlled chamber at 28 ± 2 °C and 65 ± 5% R.H. for over 10 generations, and were fed with whole wheat flour (WWF) containing 5% yeast. Two-day-old adults were utilized for subsequent experiments.

#### 2.9.2. Mortality of *S. zeamais*

After being starved for 24 h, *S. zeamais* adults were treated with different doses of *B. thuringiensis* parasporal crystals obtained with the assistance of LFU. The methods were carried out in accordance with Cui’s methods from their paper published in 2023 (with slight modifications) [7]. Briefly, *B. thuringiensis* parasporal crystals were prepared at five dose rates of 10, 20, 30, 40, and 50 mg/g wheat. Then, fifteen *S. zeamais* adults were introduced into the flasks with the aforementioned feed. The internal necks of the flasks were covered with polytetrafluoroethylene to prevent insects from escaping, and finally, the flasks were sealed with nylon gauze. The insect mortality was recorded at regular intervals (48 h) for 14 days. The mortality was calculated according to Abbott’s formula [22]. Meanwhile, the control experiments were conducted with wheat only. Each treatment was repeated three times. The lethal concentration to 50% of insects (LC_50_) and half knock-down time (KT_50_) were obtained through probit analysis.

### 2.10. Statistical Analysis

The mortality of insects in the treatments was analyzed according to the Abbott equation [22] as follows:

(2) $$x=\frac{Mortality\;in\;treatments(\%)-Mortality\;in\;control(\%)}{100-Mortality\;in\;control(\%)}\times 100\%$$

Here, *x* represents the actual mortality in the treatments. The values of lethal concentration/time to 50% of insects (LC_50_/KT_50_) were obtained through probit analysis. Data analysis was conducted using SPSS for Windows (Version 20.0; IBM Corp., Armonk, NY, USA). Statistical significance was determined using a one-way ANOVA followed by Student’s test. The results are presented as the mean ± standard deviation (SD) and were considered significant at *p* < 0.05. Images were plotted with Origin 2021 software. Particle size distribution was calculated by Nano Measurer 1.7.

## 3. Results

### 3.1. Effect of LFU on Growth of B. thuringiensis and Its Parasporal Crystal Production

**Figure 1:**
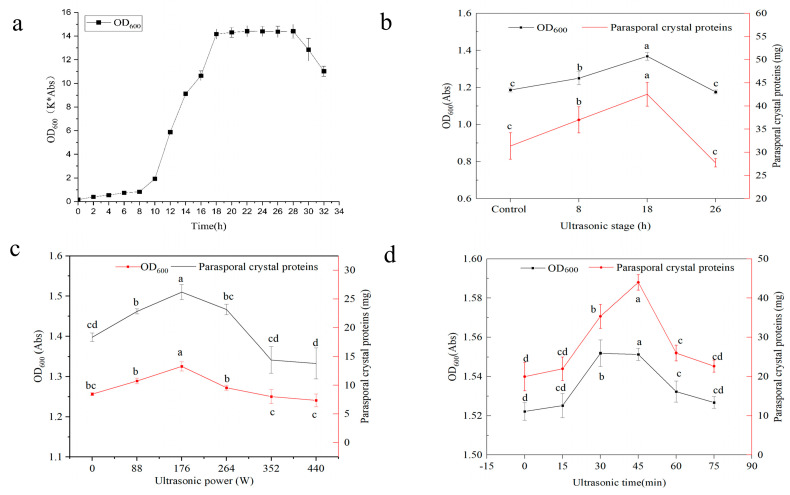
The effect of ultrasound on the growth of *B. thuringiensis* and parasporal crystal production. (**a**) The growth curve of *B. thuringiensis*. (**b**) Ultrasonic stage. (**c**) Ultrasonic power. (**d**) Ultrasonic time. Error bars indicate the standard deviations of triplicate samples. Different letters indicate significant differences at *p* < 0.05. K is a coefficient related to the instrument, Abs represents absorbance.

The effects of ultrasound with varying parameters (power, duration, and bacterial growth stage) on the biomass of *B. thuringiensis* and yields of parasporal crystals were analyzed (Figure 1). Ultrasound significantly affected the biomass of *B. thuringiensis* and its production of parasporal crystals. Sonication (40 kHz, 264 W, 45 min) was implemented following the incubation of *B. thuringiensis* for 8, 18, and 26 h, corresponding to the latent prophase, the late exponential phase, and metaphase, respectively, according to the growth curve (Figure 1a,b). Compared to the control, both the biomass of cells and the parasporal crystals obtained were significantly increased by LFU in the latent prophase and late exponential phase, but not in the metaphase, and reached their maximum values in the late exponential phase (18 h).

Then, the effect of ultrasonic power (at 0 to 440 W and 40 kHz for 45 min) on the biomass of *B. thuringiensis* in the late exponential phase and on the final parasporal crystal production was examined (Figure 1c). We found a gradual increase in the biomass of *B. thuringiensis* and its production of parasporal crystals with increasing ultrasonic power, and both reached their maximum values at 176 W and then decreased with a further increase in power. So, an ultrasound power of 176 W was selected for the subsequent experiment. The effect of ultrasonic duration (15–75 min) with a power of 176 W in the late exponential phase was determined. As shown, the biomass of *B. thuringiensis* and the parasporal crystal yields gradually increased within 15–45 min of continuous LFU treatment and then decreased with increasing exposure time (Figure 1d). The maximum values of both were obtained after 45 min of LFU treatment with a power of 176 W in the late exponential phase. Therefore, a power of 176 w for continuous 45 min of LFU treatment was regarded as the optimum ultrasonic condition to treat *B. thuringiensis* HD1 in the late exponential phase (18 h).

### 3.2. Effect of LFU on Permeability of B. thuringiensis Cell Membrane

**Figure 2:**
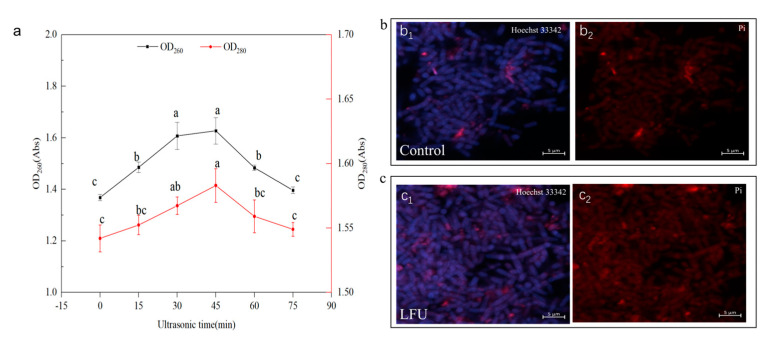
The changes in the permeability of the *B. thuringiensis* cell membrane after LFU treatment. (**a**) The OD_260_ and OD_280_ values of *B. thuringiensis* under different conditions; (**b_1_**–**c_2_**) CLSM images, including the cells stained with Hoechst 33342 and PI, respectively. The data are expressed as the mean ± SD (*n* = 3). Values followed by different letters represent statistical significance at *p* < 0.05 using ANOVA followed by Tukey’s HSD test.

The changes in cell membrane permeability could also be reflected by the OD_260_ and OD_280_ values of the bacterial culture. Ultrasound exerted a significant effect on the permeation of proteins and nucleic acids in the extracellular fluid of *B. thuringiensis* (Figure 2). The concentrations of extracellular nucleic acids and proteins gradually increased and then decreased with an increase in ultrasonic time (Figure 2a). When the ultrasonic time reached 45 min, both OD_260_ and OD_280_ increased to their maximum values, indicating that the amount of nucleic acid and protein leakage also reached the maximum. Subsequently, the amount of nucleic acid and protein leakage gradually decreased after 45 min of LFU treatment.

The ultrasound could modify the cellular structure, especially cell membrane permeability, which was further verified by a fluorescence microscope (CLSM). Hoechst 33342 and PI were used for double-staining of the *B. thuringiensis* cells. Compared to the control, the *B. thuringiensis* cells treated with ultrasound showed slightly more red with fluorescence and much more weak blue (Figure 2(b_1_–c_2_)).

### 3.3. Effect of LFU on Morphology of B. thuringiensis Cells

**Figure 3:**
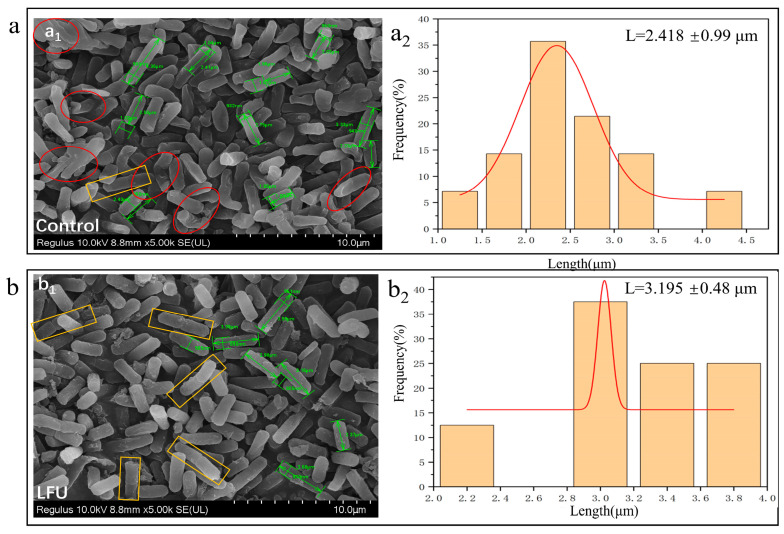
SEM (**a_1_**,**b_1_**) and cell length distribution (**a_2_**,**b_2_**) analysis of *B. thuringiensis*. (**a**) Control group; (**b**) LFU group. The red circles indicate shrunken cells, the green indicates the size of cell, and the orange boxes indicates binary fission.

The effect of LFU on the morphological characteristics of *B. thuringiensis* cells was analyzed using SEM (Figure 3). The SEM analysis indicated that *B. thuringiensis* cells without ultrasound treatment had a smooth surface, but some slightly shrank at 26th hour of the growth phase (Figure 3(a_1_)); after being cultured to 26 h, the *B. thuringiensis* treated with LFU (40 kHz, 176 W) at the 18th hour of the growth phase for 45 min exhibited slightly changed cell structures (Figure 3(b_1_)). A tiny number of cells were damaged, but some binary fission appeared, and the cells became longer compared to those of the controls; the length of *B. thuringiensis* treated with and without LFU was 2.418 and 3.195 μm, respectively (Figure 3(a_2_,b_2_)).

### 3.4. LFU Stimulates B. thuringiensis Spore Germination

**Figure 4:**
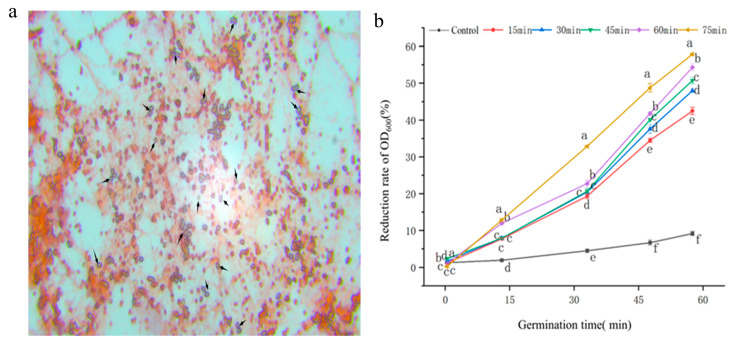
Effect of LFU on the germination of *B. thuringiensis* spores. (**a**) Staining image of *B. thuringiensis* spores in control using optical microscope. (**b**) The reduction rate of the OD_600_ value of spore suspension after LFU treatment. The data are expressed as the mean ± SD (*n* = 3). Values followed by different letters represent statistical significance at *p* < 0.05 using ANOVA followed by Tukey’s HSD test. Spores marked by arrows are un-germinated.

After staining *B. thuringiensis* spores, spore germination was investigated using a microscope (Figure 4a). Microscopic examination revealed a spore count of 4 × 10^8^ CFU/mL after cultivation, with a spore rate of 92.8%. During spore germination, the refractive index decreases gradually, leading to a gradual reduction in the OD_600_ value of the spore suspension. The spore germination was improved by increasing the duration of LFU treatment; after 75 min of ultrasonic treatment followed by 60 min of fermentation, the absorbance reduction rate was approximately 6 times that of the untreated sample (Figure 4b).

### 3.5. Effect of LFU on Inositol Concentration of B. thuringiensis Fermentation

**Figure 5:**
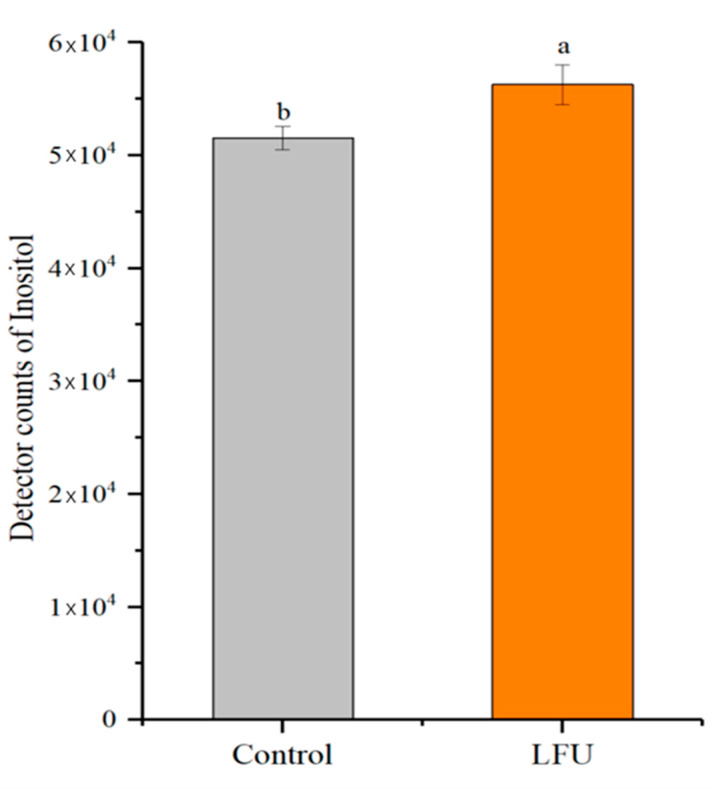
The inositol concentration of *B. thuringiensis* fermentation supernatant under different conditions. The data are expressed as the mean ± SD (*n* = 3). Values followed by different letters represent statistical significance at *p* < 0.05 using ANOVA followed by Tukey’s HSD test.

Moreover, the yields of inositol in *B. thuringiensis* culture after LFU treatment were significantly increased compared to the controls (Figure 5). This might be the reason for *B. thuringiensis* in the LFU group speeding up the development of cell and spore germination.

### 3.6. Effect of LFU on Physicochemical Characteristics of B. thuringiensis Parasporal Crystals

**Figure 6:**
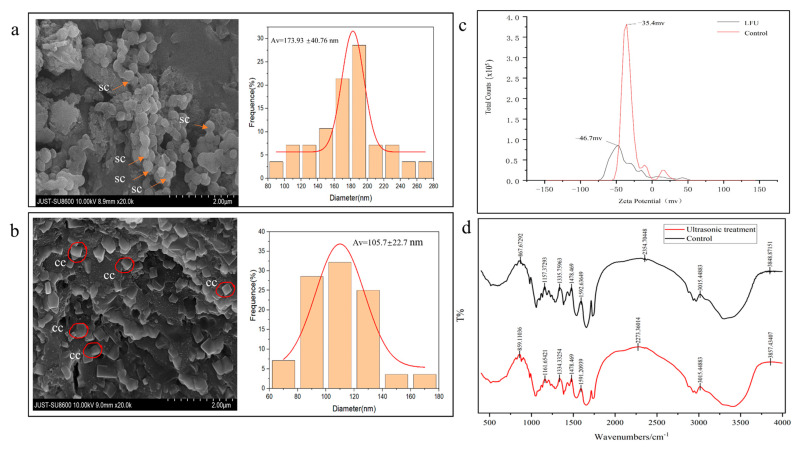
The physicochemical characteristics of *B. thuringiensis* parasporal crystals. (**a**,**b**) SEM images and particle size distribution plots of parasporal crystals obtained from the control (**a**) and LFU groups (**b**) sc, spherical crystal; cc, cuboidal crystal. (**c**) FTIR analysis and (**d**) zeta potential of the obtained parasporal crystals.

The morphological features of parasporal crystals from *B. thuringiensis* were analyzed using SEM (Figure 6a,b). Parasporal crystals obtained without FLU showed nearly spherically shaped particles with well-defined boundaries and a uniform distribution, while those in the FLU group showed a more square structure, and not sphericity. And there was significant difference in the particle sizes of the parasporal crystals in the LFU group (173.93 nm) and controls (105.7 nm). The parasporal crystals obtained in the control and LFU groups were negatively charged, and their zeta potential values were −35.4 and −46.7 mV, respectively (Figure 6c).

The changes in the functional groups of parasporal crystals from *B. thuringiensis* after LFU treatment were analyzed using FTIR in the spectral range of 500 to 4000 cm^−1^ (Figure 6d). Compared to the control, some of characteristic absorption peaks of parasporal crystals in the LFU group were changed; for example, the peaks at 3289.5, 2968.4, 1729.6, 1654.0, 1539.8, 1451.4, and 1056.0 cm^−1^ shifted to 3470.75, 2968.4, 1733.9, 1652.6, 1532.7, 1447.1, and 1046.1 cm^−1^, respectively.

### 3.7. The Pesticidal Activities of B. thuringiensis Parasporal Crystals Against S. zeamais

**Figure 7:**
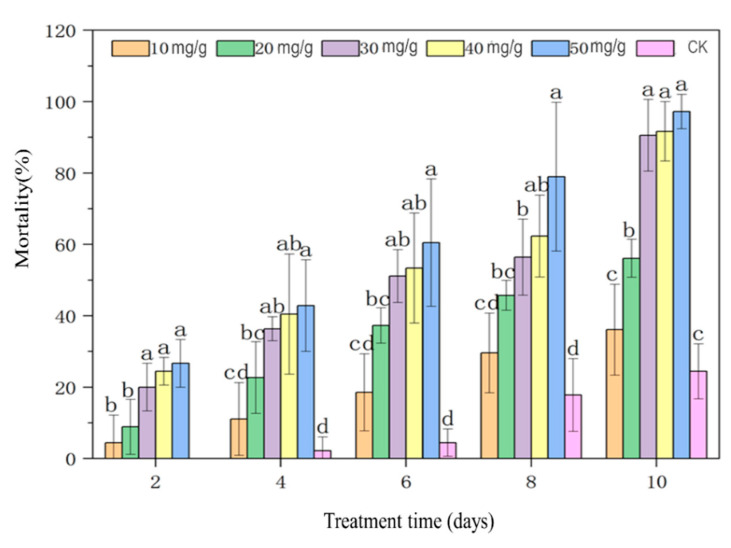
The insecticidal activities of *B. thuringiensis* parasporal crystals against *S. zeamais* adults. Values followed by different letters represent statistical significance at *p* < 0.05 using ANOVA followed by Tukey’s HSD test.

The effects of *B. thuringiensis* parasporal crystals obtained with LFU on the mortality of *S. zeamais* adults were assessed (Figure 7). The mortality of *S. zeamais* adults increased with increasing *B. thuringiensis* parasporal crystal concentrations and exposure times. 10 days of exposure to 30 mg/g of parasporal crystals led to the death of nearly 100% *S. zeamais* adults.

Based on the bioassay experiment, the toxicity regression equation of the parasporal crystals obtained was y = 4.695x − 4.851, *p* < 0.001, where y is the mortality (%) in probits, and x is the log_10_ dose. Based on this equation, the LC_50_ of parasporal crystals was 10.795 mg/g on day 14 (Table 1). When *S. zeamais* adults were treated with 30 mg/g of parasporal crystals for an increasing number of days, the mortality of the insects increased. The KT_50_ regression equation of the parasporal crystals was y = 4.494x − 3.083, *p* < 0.001, where y is the mortality (%) in probits, and x is the log_10_ time. The Kt_50_ of parasporal crystals was 4.855 days at a concentration of 30 mg/g (Table 2).

## 4. Discussions

Low-frequency ultrasound (LFU) has been proven to be effective in enhancing the growth and metabolite production of microorganisms, receiving increasing attention in recent years. Parasporal crystals, known as δ-endotoxins, comprise two families, Crystalline (Cry) and Cytolytic (Cyt) proteins, which have been proven to be specifically toxic to Coleoptera, Lepidoptera, Diptera, or Orthoptera insects [23,24]. The increase in the biomass of *B. thuringiensis* and its parasporal crystals is of great significance for its application in the agricultural field. LFU could not only improve the biomass of *B. thuringiensis* cells and stimulate their metabolism, but also alter the physicochemical characteristics of the parasporal crystals they produced. During this process, three factors, including the growth stage of bacteria, ultrasonic power, and ultrasonic treatment time, exerted significant effects on the biomass of the cells and the yields of parasporal crystals.

It is well known that microorganisms in the exponential phase are much more active and sensitive to external stimuli, resulting in rapid growth and proliferation [25]. The most significant positive effect was achieved after the LFU treatment of *B. thuringiensis* in the late exponential phase; the biomass of *B. thuringiensis* and its parasporal crystal production were significantly improved by 5.35% and 17.96%, respectively. The changes in the yields of parasporal crystals were similar to the trend of changes in the biomass of *B. thuringiensis*. These results are in agreement with those of other similar reports; for example, in studies on *B. licheniformis* YYC4 and *B. circulans* MTCC 7942, after ultrasonic treatment, both the biomass of these bacteria and their protease activity/production were remarkably increased [26,27]. Ultrasound power, the intensity of ultrasound, is another important factor affecting the growth of microorganisms. Low-intensity ultrasound (<1 W/cm^2^) can effectively facilitate the growth of microorganisms, whereas excessive sonication leads to the opposite outcome [28]. The results of this study showed that a power of LFU below 264 W positively influenced the biomass of *B. thuringiensis* and parasporal crystals yields. The optimal power level was determined to be 176 W, corresponding to a power density of 11.7 W/L, which resulted in respective increases of 5.5% and 43.7%. Low-intensity ultrasonic power can generate stable cavitation to effectively enhance metabolism and promote the growth of microorganisms, while high-intensity ultrasonic power produces more intense shock waves and strong mechanical shear forces, resulting in reversible cell damage and even cell death [27,29]. The ultrasonic time is also a crucial factor for promoting bacterial fermentation. As LFU duration increases, the biomass of *B. thuringiensis* and its production of parasporal crystals increased, reaching maximum values of 9.6% and 58.3%, respectively, at 45 min. However, overly long-term ultrasound stimulation led to their decrease. These results are consistent with those of previous reports; the biomass of *Ashbya gossypii* and its riboflavin production increased within 45 min of ultrasound treatment at 210 W and 33 kHz during an exponential phase of 48 h [30], and the growth of *B. licheniformis* YYC4 in same phase was enhanced under ultrasonic treatment at 28 kHz for 1.5 h and at 120 W for 2 h, but decreased with a further increase in duration [27]. These differences may be attributed to the diverse cellular structures of microorganisms and their varied responses to ultrasound stimulation. Appropriate ultrasonic treatment is of considerable significance for promoting microbial fermentation efficiency. Herein, favorable ultrasound with a power of 176 W and a frequency of 40 kHz was employed for 45 min to stimulate *B. thuringiensis* during a late exponential phase of 18 h.

Nucleic acids and proteins had strong peaks at 260 nm and 280 nm, respectively. After 45 min of ultrasonic treatment, the permeability of the *B. thuringiensis* cell membrane was significantly enhanced, resulting in increased leakage of nucleic acids and proteins from the cells. Notably, nucleic acids exhibited greater sensitivity to this treatment compared to proteins (Figure 2). This observation is akin to the findings of a study regarding *B. licheniformis* YYC4, which demonstrated an increase in extracellular protein and nucleic acid levels following treatment with LFU [27].

The physical characteristics of *B. thuringiensis* cells and the parasporal crystals obtained were examined. The results of the FESEM/CLSM analysis showed that the cells treated after LFU became longer with a higher degree of organelle dispersity, and the membranes became much thinner and more permeable; in addition, there were small pores and disruptions in the cell walls, which could further facilitate the exchange of these nutrients and metabolites [26]. It has been reported that Hoechst 33342 can bind to the DNA of all cells; healthy cells exhibit weak blue nuclei, and cells that have undergone apoptosis show strong blue nuclei. Meanwhile, PI can only bind to the DNA or RNA of necrotic cells, causing the cells to display red fluorescence [18]. The cells treated with LFU showed more red nuclei, revealing that the PI dye targeted the ultrasonic-treated cells, and a certain number of bacterial cells were apoptotic. The LFU-treated cells showed weak blue fluorescence, revealing that LFU could increase the weak destructive effect on the cell membranes, but the majority of cells were healthy. LFU could enhance the permeability of the cell membrane. After LFU treatment, the *B. subtilis* ZHX cells showed full, swollen, and significantly wider morphologies; although a tiny number of cells were damaged due to ultrasonic shock waves and mechanical shear forces, they could recover once the stimulation stopped [25].

During sporulation, parasporal crystals were simultaneously generated and released from *B. thuringiensis* cells. Following a period of cultivation, the decrease in the absorbance of the culture medium was directly proportional to the germination of *B. thuringiensis* spores. After low-frequency ultrasound treatment for a certain period of time (0~70 min), the absorbance value decreased significantly. These results correspond to those of previous studies about *B. licheniformis* YYC4, which showed that the number of spores decreased with increasing ultrasound time (0~1.5 h) [27]. So, LFU could also promote germination of *Bacillus* sp. spores, which would facilitate the control of the germination of endospores when combined with other stimulation methods, such as heat, pressure, electricity, and radiation. Inositol, an essential growth factor for cellular organisms, was found to be elevated. It has been reported that inositol can provide *Drosophila melanogaster* with sufficient catabolism to meet their basic energy requirements and facilitate adaptation to environmental stresses [31,32,33]. The increase in inositol further demonstrated the function of LFU in accelerating microbial cell growth and fermentation production, although the details of the mechanism still require further study.

Due to the multiple 3D structures of Cry proteins, parasporal proteins often exhibit different crystal types (i.e., bipyramid, round, abnormal, mosaic, and spherical crystals) and biological toxicity against different insect species [1,34,35,36]. SEM analysis showed that most of the parasporal crystals produced by *B. thuringiensis* subsp. *kurstaki* HD1 were bigger, spherical, and irregularly shaped, while those obtained with LFU treatment were square and smaller. It has been reported that the parasporal crystals of *B. thuringiensis* subsp. *kurstaki* HD1 usually contain one bipyramidal crystal and one ovoid inclusion [1]. Most insecticides formulated with *B. thuringiensis* subsp. *kurstaki* HD1 are toxic to Lepidoptera, while other products toxic to Coleoptera and Diptera are formulated with *B. thuringiensis* subsp. *tenebrionis* [35]. The alteration in the 3D structure of crystal protein might affect its insecticidal characteristics. For example, parasporal crystals produced by *B. thuringiensis* S3076-1 were mainly spherical and irregularly shaped, and exhibited strong toxicity to Lepidoptera insects, such as *Spodoptera frugiperda* and *Helicoverpa armigera* [36].

The FT-IR spectral analysis showed that the protein secondary structure of the crystals also changed due to LFU. The absorption band ranging from 3289.5 to 3470.75 (Amid A) is attributed to the stretching vibration of the N-H bond. The increase in wave number is in accordance with the fact that the frequency of stretching vibration is reduced due to hydrogen bond formation. This could be ascribed to the disruption of hydrogen bonds in sonication by means of an increase in the net negative charge, which is consistent with the zeta potential results. A 31.9% increase in the net negative surface charge of the crystal produced with the assistance of LFU treatment was demonstrated. These results are in correspondence with the research of Nazari [37]; the bands at 2968.4 to 2929.42 cm^−1^ could correspond to CH_2_ stretching vibrations. The bands at 1729.6 and 1733.9 cm^−1^ indicate the existence of C=O Stretching. Spectral bands at 1654.0 and 1652.6 cm^−1^ (amide I band) correspond to the C-O stretching vibrations of protein linkages, which are most sensitive to alterations in the protein secondary structures of α-helix compared to the amid II regions (1480–1575 cm^−1^). Bands at 1539.8 and 1532.7 cm^−1^ correspond to NO₂ stretching. The decrease in wave number might be attributed to the transformation of a portion of the random coil into a β-sheet in the crystal. An alteration in the secondary structure of the crystal was revealed by the positional change in the amid II region. These results are in agreement with these of previous studies [36,37]. The peaks at 1451.4 and 1447.1 cm^−1^ might correspond to CH_2_ stretching. The peaks at 1056.0 and 1046.1 cm^−1^ could correspond to C-O stretching vibration. The peak at 512.3 cm^−1^ is attributed to unsaturated bonds [38]. Owing to its capillary surface waves and acoustic cavitation, LFU treatment could modify the secondary structure of crystal proteins by increasing the sludge content of amino functional groups and enhancing the microbial mass transfer efficiency, resulting in changes in their functional properties, such as their rheological properties, solubility, and emulsifying activity [39], the details of which will be further studied in the future.

The parasporal crystals obtained through LFU-assisted treatment exhibited good pesticide activity against *S. zeamais* adults. *S. zeamais* is a typical Coleoptera pest insect worldwide, causing serious grain losses in terms of both weight and quality [40]. The major protoxin proteins belonged to the Cry1 and Cry2 classes, which showed greater effectiveness in controlling the lepidopteran than coleopteran pests [41,42]. Meanwhile, the parasporal crystals obtained from *B. thuringiensis* HD1 with the assistance of LFU demonstrated a favorable effect against *S. zeamais*, which might be attributed to the structural changes caused by LFU. In the future, the scope of pest control by parasitoid crystal proteins will be significantly broadened, and their efficiency will also be enhanced with the application of diverse modification technologies. For instance, microencapsulated *B. thuringiensis* crystal proteins with gum arabic and maltodextrin led to higher mortality against *S. frugiperda* and *M. sexta* larvae than unencapsulated ones [23].

## 5. Conclusions

In this paper, the effect of low-frequency ultrasound (LFU) on the biological characteristics of *B. thuringiensis* cells and their production of parasporal crystals, including the growth of cells; the yields of parasporal crystals; and the morphological, structural, and insecticidal activities against *S. zeamais* adults, was investigated. Additionally, the related mechanism was further explored. Three factors, namely the growth stage of bacteria, ultrasonic power, and ultrasonic treatment duration, significantly affected the growth and metabolism of the bacteria. LFU could significantly enhance the growth of bacterial cells and the yields of parasporal crystals by increasing cell membrane permeability, the synthesis rate of inositol, and the germination rate of spores. Furthermore, the structure of parasporal crystals obtained with LFU assistance was more regular and uniformly distributed than the structure of those obtained without LFU treatment. The parasporal crystals obtained exhibited good insecticidal activities against *S. zeamais* adults, with an LC_50_ of 10.795 mg/g on day 14 and a Kt_50_ of 4.855 days at a concentration of 30 mg/g.

While our study further demonstrates the significant potential of low-frequency ultrasound (LFU) in enhancing microbial fermentation for target product synthesis due to its environmental friendliness and high efficiency, critical challenges persist in scaling up this technology for industrial production and practical implementation. For instance, the efficacy of ultrasonic treatment exhibits marked heterogeneity across microbial species, posing significant challenges in processing standardization. Additionally, the substantial capital expenditures and operational expenditures associated with industrial-scale ultrasonic systems may constrain their commercial viability. Furthermore, ultrasonic exposure may induce unintended alterations in microbial metabolic flux and transcriptional regulation patterns, necessitating rigorous validation of its consistency and biosafety profiles and the stability of its production. To date, the relationship between the growth of *B. thuringiensis* cells in the presence of LFU and the regulation mechanism of inositol synthesis has not been previously reported, providing new research avenues for future studies. In addition, the stability of parasporal crystals obtained with LFU assistance and their function will be further examined. The technological development of LFU in *B. thuringiensis* fermentation for synthesizing parasporal crystals is necessary to broaden its application in the field of insect pest control.

## Figures and Tables

**Table 1:** Mortality of *S. zeamais* adults exposed to different concentrations of *B. thuringiensis* parasporal crystal proteins.

Con (mg/kg Flour)	7-day Mortality (%) ± SD	Slope Function (Slope)	LC_50_ ± SE (mg/kg)	LCL–UCL (mg/kg)	χ^2^
Control	24.44 ± 7.70 e	−4.851 ± 1.003	10.795	7.718–13.314	12.808 *
10	36.11 ± 12.73 c				
20	56.11 ± 5.36 b				
30	90.56 ± 10.05 a				
40	91.67 ± 8.33 da				
50	97.22 ± 4.81 a				

Means followed by different letters are significantly different (*p* < 0.05; one-way ANOVA and Tukey’s test). SD, standard deviation; SE, standard error; Con, concentration; LCL, lower confidence limit; UCL, upper confidence limit; χ^2^, Chi-square test. *, there is significant difference in the content of *B. thuringiensis* parasporal crystal proteins to the mortality of insects after Chi-square test (*p* < 0.05).

**Table 2:** Mortality of *S. zeamais* adults exposed to 30 mg/g of *B. thuringiensis* parasporal crystal proteins for different durations.

Exposure Time (days)	Mortality (%) ± SD	Slope Function (Slope)	KT50 ± SE (days)	LCL–UCL (days)	χ^2^
0	0.00 e	−3.083 ± 0.534	4.855	4.075–5.657	11.718 *
2	20 ± 6.67 d				
4	36.35 ± 3.38 cd				
6	51.11 ± 7.40 bc				
8	56.42 ± 10.62 b				
10	90.56 ± 10.05 a				

Means followed by different letters are significantly different (*p* < 0.05; one-way ANOVA and Tukey’s test). SD, standard deviation; SE, standard error; Con, concentration; LCL, lower confidence limit; UCL, upper confidence limit; χ^2^, Chi-square test. *, there is significant difference in the content of *B. thuringiensis* parasporal crystal proteins to the mortality of insects after Chi-square test (*p* < 0.05).

## Data Availability

The data are contained within the article.

## Abbreviations

The following abbreviations are used in this manuscript:

LFU	Low-frequency ultrasound
FESEM	Field emission scanning electron microscopy
CLSM	Laser confocal scanning electron microscope
FTIR	Fourier transform infrared
LC50	Lethal concentration to 50%
KT50	Half knock-down time
SD	Standard deviation
SE	Standard error
Con	Concentration
LCL	Lower confidence limit
UCL	Upper confidence limit
χ2	Chi-square test
Cry	Crystalline
Cyt	Cytolytic
